# Spontaneous Thrombosis of a Long-Segment Hepatic Artery Aneurysm During Conservative Management: A Case Report on an 11-Year Follow-Up

**DOI:** 10.7759/cureus.105287

**Published:** 2026-03-16

**Authors:** Hiroki Higashihara, Hiroki Satomura, Takayasu Yamamoto, Yasushi Kimura, Kaishu Tanaka, Yusuke Ono, Noriyuki Tomiyama

**Affiliations:** 1 Department of Radiology, The University of Osaka Hospital, Suita, JPN; 2 Department of Diagnostic and Interventional Radiology, The University of Osaka Graduate School of Medicine, Suita, JPN; 3 Department of Radiology, Sumitomo Hospital, Osaka, JPN; 4 Department of High Precision Image-Guided Percutaneous Intervention, The University of Osaka Graduate School of Medicine, Suita, JPN

**Keywords:** celiac artery dissection, collateral circulation, conservative management, dissecting aneurysm, hepatic artery aneurysm, long-segment aneurysm, long-term follow-up, mural thrombus, spontaneous thrombosis, visceral artery aneurysm

## Abstract

Hepatic artery aneurysms (HAAs) can rupture, yet the management of small, asymptomatic, long-segment lesions remains controversial. We report a 64-year-old man with an incidentally detected, long-segment aneurysmal change extending from the proximal common hepatic artery to the distal proper hepatic artery (approximately 80 mm). CT showed irregular, multilobulated ectasia with a focal saccular component (17 mm), an intimal flap, and mural thrombus, suggesting aneurysmal degeneration after dissection; a subtle dissection-like change was also suspected in the celiac trunk. Given the small maximal diameter, absence of symptoms, and concern for hepatic/biliary ischemia with intervention, close imaging surveillance was chosen. Progressive thrombosis led to complete occlusion at 5.5 years, with preserved hepatic enhancement and a more prominent left-gastric-right gastric collateral pathway. The aneurysm remained stably thrombosed without ischemic complications over 11 years. This case highlights that carefully selected, asymptomatic, anatomically complex HAAs may be managed conservatively with strict long-term follow-up.

## Introduction

Hepatic artery aneurysms (HAAs) are the second most common visceral artery aneurysms after splenic artery aneurysms, and are clinically important because rupture is associated with high mortality [1-5]. With the increasing use of cross-sectional imaging, visceral artery aneurysms, including HAAs, are more frequently identified incidentally [5-7].

Current practice generally favors intervention for symptomatic aneurysms, rapidly enlarging lesions, pseudoaneurysms, and aneurysms above commonly used size cutoffs; however, the management of small, asymptomatic aneurysms, particularly fusiform or long-segment lesions, remains controversial [6-9]. Long-segment aneurysms that involve the common and/or proper hepatic artery may be technically challenging to treat, and exclusion or parent artery sacrifice may pose a risk of hepatic ischemia or biliary complications if collateral pathways are inadequate [9-12]. Given the limited data on the natural history of long-segment HAAs and the absence of robust criteria for conservative management, individualized treatment strategies based on aneurysm morphology, symptom status, patient risk factors, and perfusion anatomy are essential [4,6-7]. Here, we report a long-segment HAA managed conservatively that underwent spontaneous thrombosis during long-term follow-up without ischemic complications.

## Case presentation

A 64-year-old man with hypertension, diabetes mellitus, and atherosclerotic disease underwent routine abdominal computed tomography (CT) as part of a health screening, which incidentally revealed an aneurysmal dilatation of the hepatic artery. He had no abdominal pain, bleeding, or history of hepatobiliary surgery or trauma, and physical examination was unremarkable. Liver function tests were within normal limits.

**Figure 1:**
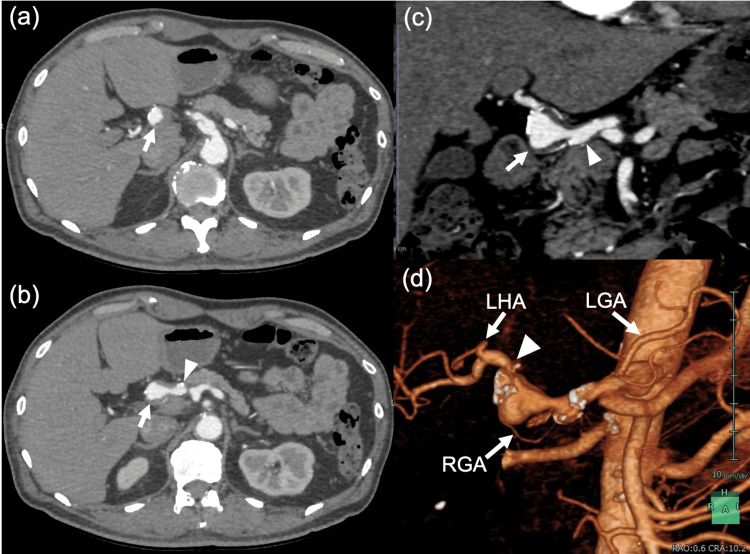
Initial contrast-enhanced CT and volume-rendered images at diagnosis (a, b) Axial arterial-phase contrast-enhanced CT images (b is 5 mm caudal to a). (c) Coronal arterial-phase contrast-enhanced CT image. An irregular long-segment aneurysmal ectasia is seen from the common hepatic artery to the proper hepatic artery. A focal saccular component measuring up to 17 mm in maximum diameter is seen (arrow). A dissection-related change is suspected immediately proximal to the saccular component (arrowhead), and mural thrombus is visible within the saccular component. Dissection-related change is also suspected in the celiac trunk. (d) Volume-rendered three-dimensional reconstruction from the same CT dataset. The image demonstrates the long-segment aneurysmal morphology and its anatomic extent from the common hepatic artery to the proper hepatic artery. The left hepatic artery (LHA) branches immediately distal to the long-segment aneurysmal segment. A collateral pathway from the left gastric artery (LGA) to the right gastric artery (RGA) is suggested, and the RGA appears to join the distal proper hepatic artery near the margin of the saccular component (arrowheads).

Contrast-enhanced CT demonstrated aneurysmal changes involving the hepatic arterial axis from the proximal common hepatic artery (CHA) to the distal proper hepatic artery (PHA), with an overall lesion length of approximately 80 mm (Figures 1a-1c). Rather than a single smooth fusiform dilatation, the involved segment showed irregular, multilobulated aneurysmal ectasia with a “beaded” appearance and a focal saccular component measuring up to 17 mm in maximal diameter. An intimal flap was identified within the proximal CHA, consistent with arterial dissection, and mural thrombus was present within the saccular component. In addition, a subtle dissection-like change was suspected in the celiac trunk around the origins of the left gastric and splenic arteries. Collectively, these findings suggested a dissecting aneurysm or aneurysmal degeneration after dissection rather than a simple true aneurysm. There was no evidence of rupture, perivascular hematoma, or distal hepatic hypoperfusion.

Volume-rendered three-dimensional reconstruction suggested collateral flow from the left gastric artery to the right gastric artery, with the right gastric artery supplying the distal portion of the PHA near the margin of the saccular component (Figure 1d). Given (i) the small maximal diameter (17 mm), (ii) the long-segment dissecting/fusiform morphology involving the CHA-PHA axis, and (iii) concern that endovascular exclusion, parent artery occlusion, or surgical repair could compromise hepatic and biliary perfusion, conservative management with close imaging surveillance was selected. Follow-up contrast-enhanced CT was performed at regular intervals, typically every 6-12 months initially and annually thereafter. Antiplatelet or anticoagulant therapy was not initiated at diagnosis.

**Figure 2:**
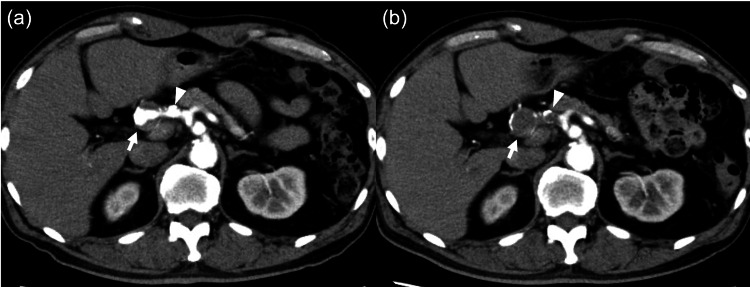
Follow-up contrast-enhanced CT at 5 years and 5.5 years (a) Arterial-phase contrast-enhanced CT image obtained 5 years after the initial diagnosis. The overall aneurysm size remains largely unchanged; however, increased intraluminal thrombosis is observed compared with baseline (arrows). The dissection-related change in the proximal segment (arrowhead) appears largely unchanged compared with the initial examination. (b) Arterial-phase contrast-enhanced CT image obtained 6 months later (5.5 years after the initial diagnosis). Complete thrombosis of the aneurysmal lumen is demonstrated (arrows). The previously identified dissection-related proximal segment is also thrombosed on this examination (arrowhead). Hepatic parenchymal enhancement is preserved, suggesting adequate collateral arterial perfusion.

Serial follow-up CT demonstrated a gradual progression of intraluminal thrombosis (Figure 2a). At 5.5 years after the initial diagnosis, complete thrombosis of the aneurysmal lumen was confirmed (Figure 2b). Despite occlusion of the hepatic artery aneurysm, hepatic parenchymal enhancement remained preserved, suggesting adequate collateral arterial perfusion. Notably, the collateral pathway from the left gastric artery to the right gastric artery appeared more prominent at the time of confirmed occlusion than at initial diagnosis, consistent with compensatory collateral development. The patient remained asymptomatic without clinical or imaging evidence of hepatic infarction or biliary ischemic complications.

**Figure 3:**
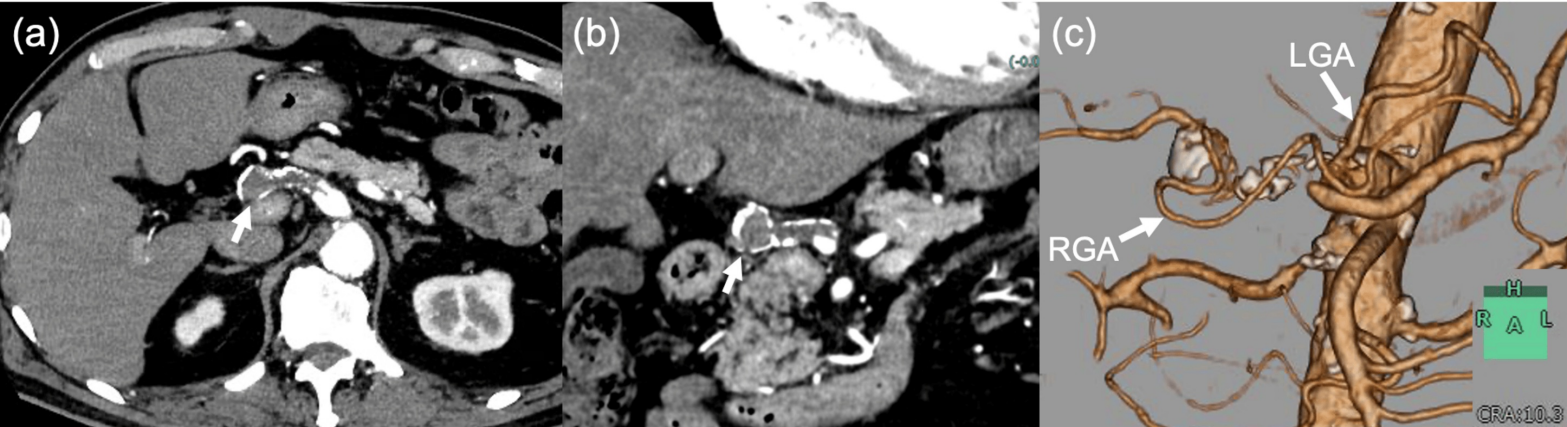
Long-term follow-up contrast-enhanced CT at 11 years (a) Axial arterial-phase contrast-enhanced CT image. (b) Coronal arterial-phase contrast-enhanced CT image. (c) Volume-rendered three-dimensional reconstruction. At 11 years after the initial diagnosis, persistent complete thrombosis of the hepatic artery aneurysm is confirmed (arrows), with no evidence of recanalization, enlargement, or secondary complications. On volume-rendered imaging, the collateral pathway from the left gastric artery to the right gastric artery is clearly visualized and appears more prominent than at the initial examination.

One year after confirmation of complete aneurysm thrombosis, antiplatelet therapy was started for newly diagnosed vertebral artery stenosis. The thrombosed HAA remained stable without recanalization or enlargement on subsequent surveillance imaging. At the most recent follow-up, 11 years after initial diagnosis, contrast-enhanced CT confirmed persistent complete thrombosis without secondary complications, and the patient remained asymptomatic (Figures 3a-3c).

## Discussion

Management of HAAs is guided by symptoms, aneurysm type (true vs pseudoaneurysm), size, growth, and anatomic feasibility of repair [5-9]. Contemporary society guidelines for visceral artery aneurysms recommend repair of symptomatic HAAs and pseudoaneurysms regardless of size, and consideration of repair for asymptomatic true HAAs beyond a diameter threshold (commonly 20 mm in several guidance documents), whereas the more recent European Society for Vascular Surgery (ESVS) guideline suggests a higher threshold (e.g., ≥30 mm) for asymptomatic HAAs [7-9]. Although size thresholds are often used to guide treatment of asymptomatic true HAAs, their applicability to long-segment dissection-related aneurysmal lesions is less certain. In such cases, lesion morphology, suspected hemodynamics, and collateral perfusion may be more relevant than diameter alone. In our case, the maximum diameter was 17 mm, the patient was asymptomatic, and the lesion was long-segment and fusiform with an associated dissection flap, making endovascular exclusion or surgical reconstruction potentially complex with a non-trivial risk of compromising hepatic arterial inflow.

Long-segment fusiform aneurysms spanning the common and proper hepatic arteries can be challenging to treat with a stent-graft because suitable proximal and distal landing zones may be limited, critical branch vessels may originate from the diseased segment, and marked angulation or tortuosity can further hinder device delivery and accurate deployment [10,11,13]. Coil embolization with parent artery sacrifice is an alternative, but its safety depends on the presence of a reliable collateral supply to the liver and bile ducts [10,11]. In the present case, although pre-treatment CT suggested developing collateral arterial pathways, their functional sufficiency was uncertain, and prophylactic parent artery occlusion was considered to carry a non-negligible risk of hepatic or biliary ischemia. Therefore, conservative management with strict imaging surveillance was selected.

Spontaneous thrombosis of visceral artery aneurysms is uncommon but has been reported, particularly in settings of altered flow dynamics, pre-existing mural thrombus, or intimal injury such as dissection [14,15]. In our patient, progressive mural thrombosis was observed during follow-up, ultimately leading to complete occlusion of the aneurysmal lumen. The combination of dissection and mural thrombus may have promoted progressive flow stagnation and thrombus propagation, culminating in complete thrombosis at 5.5 years [14,16-18].

Importantly, despite complete occlusion of the hepatic artery, no hepatic infarction or biliary ischemic complications occurred during long-term follow-up. This favorable outcome likely reflects adequate collateral arterial inflow and suggests that hepatic perfusion can be preserved even after hepatic artery occlusion in selected patients.

Although this case does not imply that conservative management should replace intervention in general, it suggests that selected asymptomatic patients with anatomically complex hepatic artery aneurysms may be safely managed with careful imaging surveillance when procedural risks are substantial and collateral perfusion appears adequate. Individualized treatment decisions based on aneurysm morphology, symptom status, and perfusion anatomy remain essential.

## Conclusions

We report a rare case of spontaneous thrombosis of a long-segment hepatic artery aneurysm managed conservatively over 11 years. In carefully selected asymptomatic patients with anatomically complex aneurysms, observation with close imaging follow-up may be a reasonable management option when interventional risks outweigh potential benefits.
